# Variable Solitary Fibrous Tumor Locations: CT and MR Imaging Features

**DOI:** 10.1097/MD.0000000000003031

**Published:** 2016-04-01

**Authors:** Ma Zhanlong, Shi Haibin, Fang Xiangshan, Song Jiacheng, Ni Yicheng

**Affiliations:** From the Department of Radiology (MZ, SH, SJ), the First Affiliated Hospital of Nanjing Medical University; Department of Pathology (FX), Nanjing Drum Tower Hospital of Nanjing University Medical School, Nanjing, Jiangsu China; and Department of Radiology (NY), University Hospitals, University of Leuven, Herestraat, Leuven, Belgium.

## Abstract

The aim of the study is to describe the radiological imaging features of different solitary fibrous tumors (SFTs) locations and present histopathological correlations.

From 2007 to 2013, 20 cases of histologically confirmed that SFTs were retrospectively analyzed with computed tomography (CT; 9/20), magnetic resonance imaging (MRI; 5/20), or both CT and MRI (6/20).

All 20 SFTs were well defined, lobular, soft-tissue masses, and 60% were located outside of the pleura. One pleural case invaded to the 10th thoracic vertebra and had lung metastases. Images revealed 11 heterogeneous lesions that exceeded 3.0 ± 0.203 cm along the greatest axis with patchy necrotic foci, and 9 homogeneous lesions <3.0 ± 0.203. Microscopically, all SFTs were proliferative spindle cells with varying degrees of fibrosis and interspersed vessel branching. Cells were strongly immunopositive for CD34.

Here we review variable imaging findings of SFTs, which can be within the pleura as well as within other serosal tissues such as the meninges and postperitoneum. SFTs > 3.0 ± 0.203 cm along the greatest axis appeared to be mixed patterns, whereas SFTs < 3.0 ± 0.203 cm had isodense appearances. SFTs cells were CD34 immunopositive and surgery was a first-line treatment choice.

**KEY POINTS**

- We depict variable imaging finding of SFTs.
- 60% of SFTs are not within pleura but in other locations.
- SFTs > 3.0 ± 0.203 cm to be mixed patterns.
- SFTs < 3.0 ± 0.203 cm had isodense appearances.
- SFTs cells were CD34 immunopositive.

## INTRODUCTION

Solitary fibrous tumors (SFTs) are rare mesenchymal tumors that typically originate from the pleura and pelvis.^1,2^ Histopathologic characteristics of SFTs were first described by Klemperer and Rabin in 1931 as pleura-localized fibrous mesotheliomas.^3,4^ Reports suggest that SFTs also originate from extrapleural sites such as the pelvis, abdomen, retroperitoneum, buccal space, maxillary sinus, liver, pancreas, suprarenal region, and kidneys.^4–10^ An SFTs diagnosis is characterized by the proliferation of spindle mesenchymal cells and positive immunohistochemical staining for CD34.^11,12^ Approximately 78% to 88% of SFTs are benign, but 12% to 22% are malignant.^13,14^ The age-standardized incidence of SFTs is reported to be 1.4 per million^15^ and their rarity, diverse localizations, and difficulty of predicating tumor biological behaviors have limited the attempts to obtain CT and MRI data to better characterize their features. Thus, more and larger population studies with CT and MRI are required to enrich our understanding on this tumor type and its behavior and to improve diagnosis and management. Here, we report data from 20 patients with both benign and aggressive SFTs at various sites.

## MATERIALS AND METHODS

From our hospital's pathology database, we identified patients diagnosed with SFTs between January 2007 and June 2013. All 20 patients with SFTs who underwent preoperative CT or MRI were included in this study. An institutional review board exemption and a waiver for the requirement of written informed consent were obtained to perform this retrospective study. Of the 20 patients, 9 had been examined with computed tomography (CT), 5 had undergone magnetic resonance imaging (MRI), and 6 had both CT and MRI.

CT imaging was performed using a 128-multidetector row scanner (SOMATOM Definitions AS 128, Siemens). Scan parameters were as follows: 5-mm slice thickness reconstructions, 36 × 36 cm field-of-view, 120 kV voltages, 100 to 220 mA current, and a 512 × 512 matrix. Contrast-enhanced CT scans were obtained at 30 and 60 s after contrast agent (Omnipaque, 300 mgI/mL, 100 mL) injection.

MRI was performed using a 3.0T MRI (Verio 3.0T, Siemens) or a 1.5T MRI unit (Gyroscan Intera; Philips Medical Systems, Best, The Netherlands). The MRI protocol included axial T1-weighted imaging (T1WI) sequences; axial and sagittal T2-weighted imaging (T2WI) sequences; and contrast-enhanced axial, sagittal, and coronal T1WI sequences (fat suppressed). An intravenous dose of 0.1 to 0.2 mmol/kg of contrast agent (gadolinium-DTPA, Schering, location) was administered to patients undergoing contrast-enhanced MR scanning.

All medical records from 20 patients were reviewed and patient age, sex, initial symptoms, treatment, follow-up imaging, and outcome were registered. CT and MRI images were reviewed independently by 2 experienced radiologists. Disagreements over imaging findings were resolved by debate, discussion, and consensus between the 2 radiologists. Imaging features of each scan were recorded for lesion location, shape, size, and number, intensity of unenhanced and contrast-enhanced lesions and classified as hypo-, iso-, or hyperintense with respect to the adjacent tissues.

Of the 20 cases, 1 had a biopsy and no additional intervention; 5 subjects underwent surgery; and 14 had biopsies before surgery. All pathologic specimens were reviewed and described by 2 experienced pathologists. Histological techniques included staining with hematoxylin and eosin (H&E) and immunohistochemical analyses including staining for CD34, CD99, B-cell lymphoma protein 2 (Bcl-2), ki67, vimentin, S-100 protein, cytokeratin (CK), and smooth muscle actin (SMA).

Numerical data such as the size of tumor at CT or MRI measured at the contrast-enhanced images of each transaxial section were averaged for each patient and descriptively compared among patients. We used the receiver operating characteristic (ROC) curve to determine the best cut-off values of the standard for sensitivity and specificity imaging features of SFTs. All descriptive and statistical analyses were performed using the Statistical Package for the Social Sciences (SPSS version 16.0; Chicago, IL). Variables are presented with standard errors (SE) and high-quality authentic CT and MRI scans and histopathological findings are presented.

## RESULTS

### Clinical Data

We included 20 subjects (12 men; mean age 43.2 years; range 31–71 years). Of the 8 patients with pleural SFTs (40%), 3 had chest pain; 4 patients were admitted for routine check-ups; and 1 patient had spine pain. Sixty percent of SFTs were located beyond the pleura and 3 brain SFTs occurred in patients who complained of dizziness and an unstable gait. Then, 3 renal SFTs were found and 1 was located in the posterior peritoneal are and 2 were pancreatic SFTs identified during a routine check-up. One pelvic SFT occurred in a subject who had urinary frequency, and 1 esophageal SFT was identified in a patient who had difficulty swallowing. Finally 1 orbital SFT was identified in a patient with vision loss.

### Imaging Findings

**FIGURE 1:**
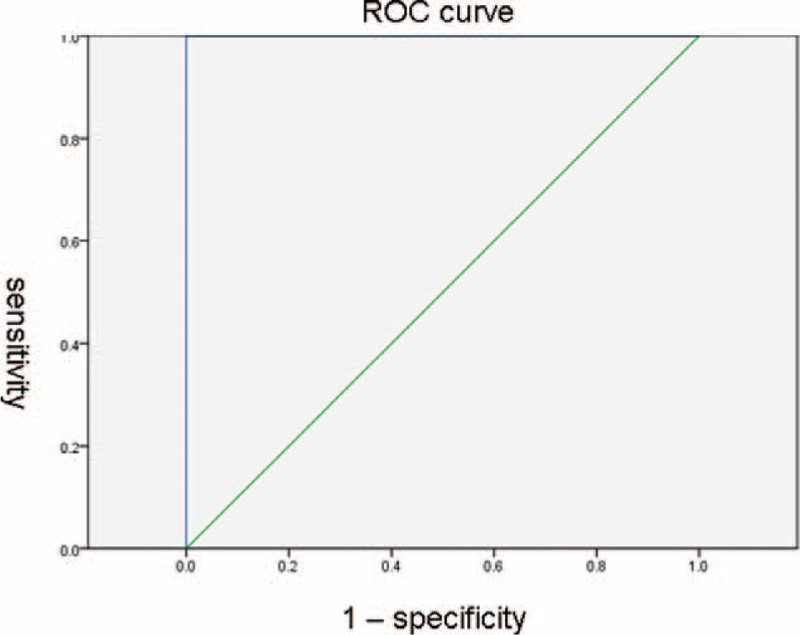
Receiver operating characteristic (ROC) curve. The SFTs imaging features were classified with homogeneous and heterogeneous enhancement. The ROC curve showed in 3.0 ± 0.203 cm of the tumor great size; the homogeneous and heterogeneous enhancement sensitivity and specificity were 0.95, respectively. ROC = receiver operating characteristic.

Imaging findings all 20 subjects with SFT were as follows: 19 were well-defined, noninvasive, lobular, soft-tissue masses with displaced and compressed neighboring tissues. One malignant pleural SFT had invaded to the T10 vertebra and there were lung metastases confirmed with CT. CT and MRI images revealed heterogeneous masses with patchy, necrotic foci in 11 lesions exceeding 3.0 ± 0.203 cm along the greatest axis, and homogeneous masses in 9 lesions <3.0 ± 0.203 cm along the greatest axis. The area under the receiver operating characteristic (ROC) curve calculated using the greatest axis of SFTs is shown in Figure 1 for each of the cohorts and for the overall patient sample, in which the area under the curve (AUC) was 0.95 (95% confidence interval, 2.98–3.03).

**FIGURE 2:**
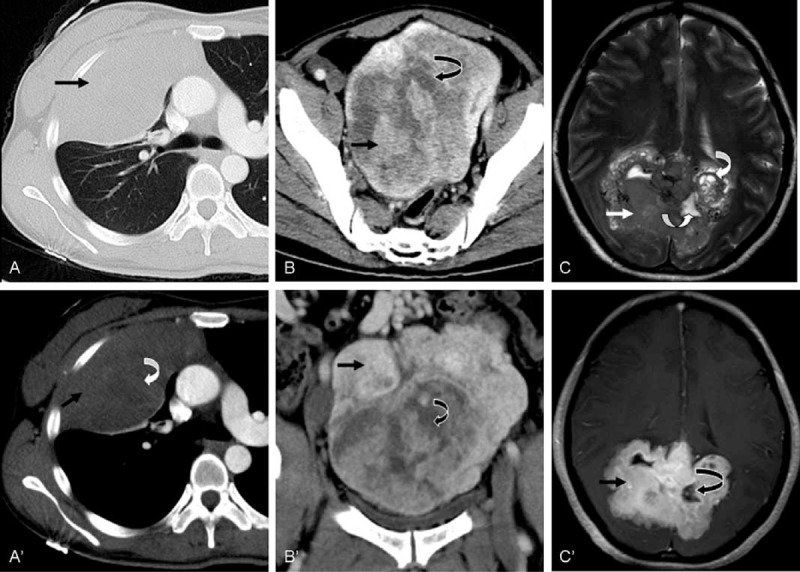
Typical features of larger SFTs. A, A’: A 35-year-old man with an incidental finding of SFT. Parenchymal (A) and mediastinal (A’) contrast material-enhanced CT images showed a round solitary fibrous tumor of the pleura. B, B’: A 54-year-old male presented with a pelvic SFT. Contrast-enhanced CT image (B, axial) and (B’ coronal) views reveal an oval, well-defined heterogeneous mass with patchy hyperdense (tumor tissue, arrow) and hypodense (necrotic tissue, curved arrow) areas and marked heterogeneous enhancement. C, C’: A 41-year-old female patient with a brain SFT. MR T2-weighted images revealed a heterogeneous hypo- and hyperintense mass in the occipital lobe (C). Contrast-enhanced T1-weighted images showed heterogeneous enhancement of the lesion (C’). CT = computed tomography, MR = magnetic resonance, SFTs = solitary fibrous tumors.

**FIGURE 3:**
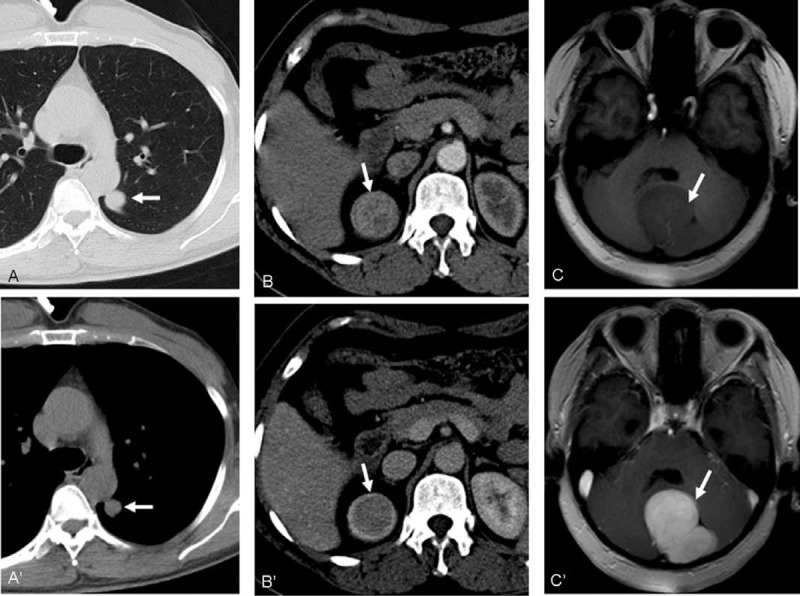
Typical features of small SFTs. A, A’: A 43-year-old man with parenchymal (A) and mediastinal windows (A’) of the CT scan showed a round solitary fibrous tumor of the pleura (≤2.8 × 2.3 cm). B, B’: A 48-year-old female patient presented with a right kidney solitary fibrous tumor. Arterial (B) and portovenous (B’) phases of contrast-enhanced CT showed a spherical, well-defined homogeneous mass with marked homogeneous enhancement. C, C’: A 68-year-old female patient with brain SFT. A T1-weighted axial MRI scan revealed a large lobulated hypointense mass (arrow) in the left cerebellum (C). Gadolinium-enhanced T1-weighted axial MRI (C’) showed marked and homogeneous enhancement of the mass (arrow). CT = computed tomography, MRI = magnetic resonance imaging, SFTs = solitary fibrous tumors.

For the larger SFTs, enhancement after contrast injection was typically intense and heterogeneous with central areas of low attenuation that correlated with necrosis, hemorrhage, or cystic degeneration. These SFTs are well-delineated and lobulated soft tissue masses (Figure 2). Smaller masses were chiefly intense and homogeneous after contrast medium enhancement. Solid masses with smoothly tapering margins were highly characteristic of these tumors (Figure 3) and calcification was not observed in any SFT in this study.

**FIGURE 4:**
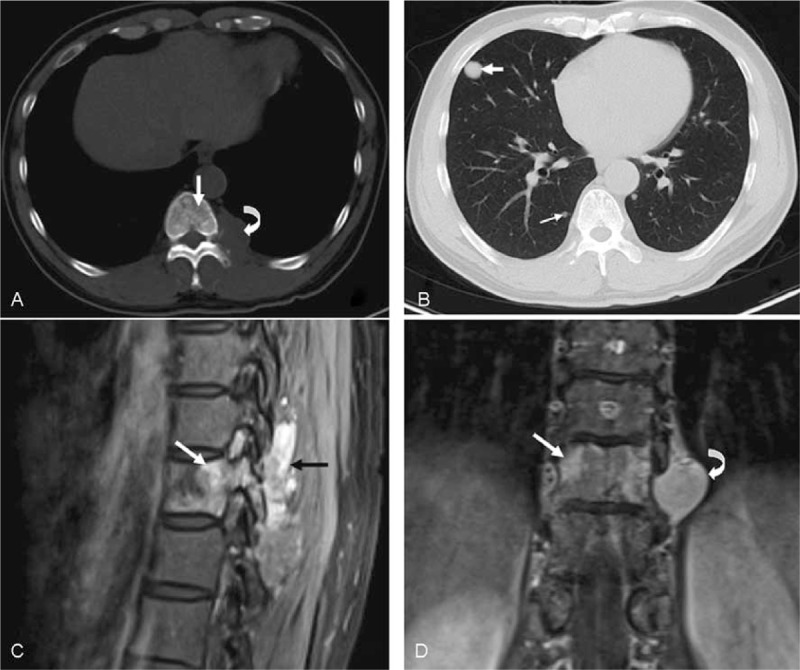
Typical features of malignant SFTs. A mass was found that originated from the pleura and was metastasized to the lung. CT mediastinal windows (A) showed a homogeneous lesion (curved arrow) in the left pleura invading to the neighboring T10 vertebral body (arrow, confirmed pathology). Parenchymal windows (B) showed metastases (arrows) in the right lung. Lesions (arrows) are marked and heterogeneously enhanced on T1-weighted MRI scans (C and D). CT = computed tomography, MRI = magnetic resonance imaging, SFTs = solitary fibrous tumors.

One case of pleural malignant SFT was well-defined and solid, 3.2 × 2.6 cm in diameter on CT scans. The tumor originated from the pleura and spread to the T10 vertebra and had metastatic foci in the lung. There were no mediastinal lymph node swelling and no liver metastases (Figure 4).

**FIGURE 5:**
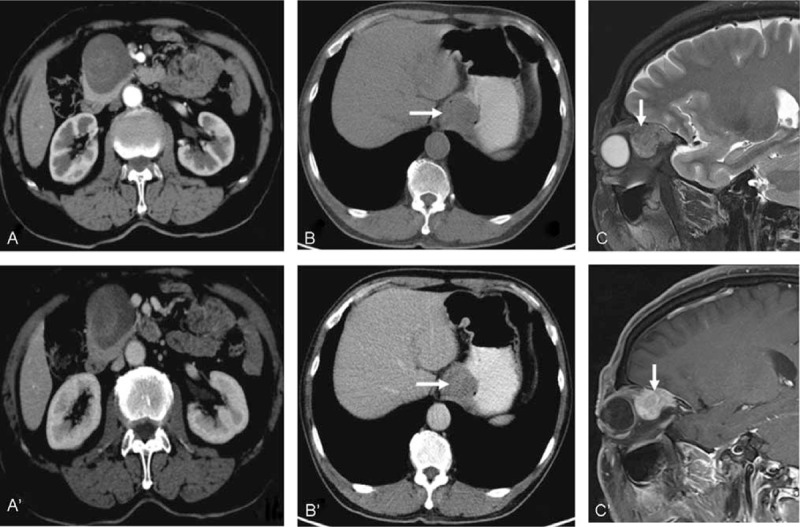
Typical features of rare locations of SFTs. A, A’: A 71-year-old man with a pancreatic SFT. Contrast-enhanced CT showed an oval, well-defined heterogeneous mass (arrows) with marked heterogeneous enhancement (A, A’). B, B’: A 69-year-old man with an esophageal SFT. CT images before (B) and after (B’) contrast-enhancement showed an oval, well-defined homogeneous mass with marked homogeneous enhancement (arrow). C, C’: A 57-year-old male patient with an orbital SFT. T1-weighted MRI scans before (C) and after (C’) contrast-enhancement showed an oval, well-defined heterogeneous mass (arrow) with marked heterogeneous enhancement. CT = computed tomography, MRI = magnetic resonance imaging, SFTs = solitary fibrous tumors.

Three patients had extremely rare SFTs of the pancreas, esophagus, and orbit (Figure 5). CT scans revealed a 4.3 × 4.6 cm, well-defined heterogeneous mass with patchy, necrotic foci in the pancreatic head. Contrast-enhanced CT images showed heterogeneous enhancement with patchy hypodense areas in both the arterial (Figure 5A) and the venous (Figure 5A’) phases with no signs of pancreatic duct obstruction or jaundice. The esophageal SFT was a 1.8 × 2.0 cm homogeneous mass located in the lower end of the esophagus (Figure 5B), which was mildly enhanced after contrast administration (Figure 5B’). For the orbital SFT, T1-weighted MRI revealed a nearly homogeneous lesion (Figure 5C), which had strong but heterogeneous contrast enhancement (Figure 5C’).

### Pathological and Immunohistochemical Findings

**FIGURE 6:**
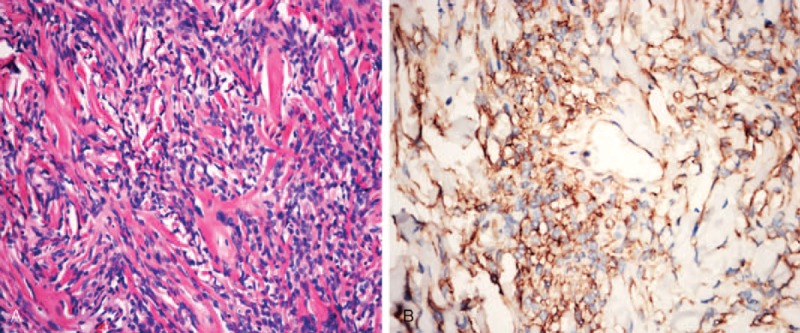
Histopathological views of SFTs. (A) Histomicrograph (H&E 200×) showed that the SFT was composed of a patternless proliferation of spindle cells with varying degrees of fibrosis. (B) A histomicrograph (200×) depicts extensive immunopositive CD34 expression. SFTs = solitary fibrous tumors.

Microscopically, all SFTs were patternless distributed proliferating spindle cells with varying degrees of fibrosis and interspersed vessel branching. There were no distinctive microscopic features, so separating SFT from other spindle cell tumors using by H&E staining alone is problematic. Immunohistochemically, SFTs were strongly immunopositive for CD34 (Figure 6). Ki67 was positively expressed in nearly 10% of SFTs and vimentin and CD99 were expressed in most SFTs. However, SFTs were typically immunonegative for Bcl-2, SMA, CKpan, S-100, and endosomal membrane (EMA) protein (Table 1).

**TABLE 1:**
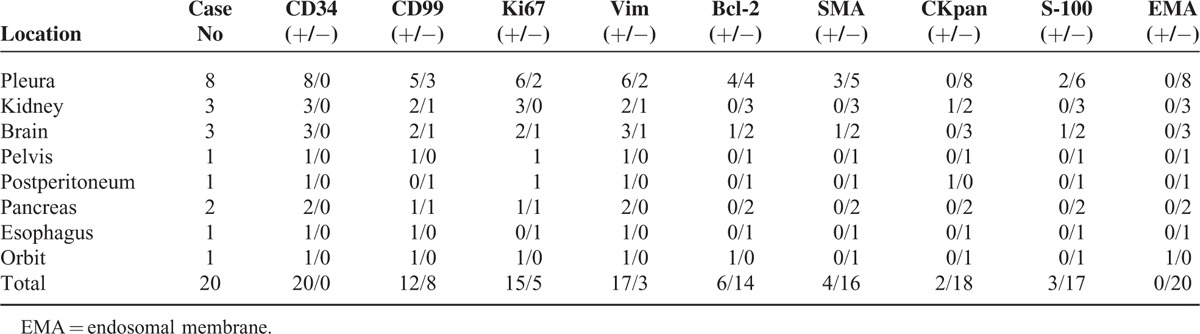
Immunohistochemistry Findings of the 20 Cases With Solitary Fibrous Tumors

## DISCUSSION

SFTs are extremely rare mesenchymal spindle cell neoplasms that most commonly occur in the pleura or other serosal surfaces and the histological origins are uncertain. Histopathologically, SFTs have a “patternless” architecture characterized by the coexistence of hypo- and hypercellular areas separated by fibrous stroma having “hemangiopericytoma-like” branching blood vessels. A final diagnosis is often made with immunohistochemical findings. Immunopositivity for CD34 is the key to SFTs diagnosis and positive expression of vimentin; CD99 and Bcl-2 occur in most SFTs. However, cytokeratin, α-SMA, and S-100 are usually absent.^11,12^

Submesothelial cells can differentiate into mesothelial cells, so SFTs occur at often-overlooked sites.^16^ In this study, we identified 20 patients diagnosed with SFTs between January 2007 and June 2013 and of these subjects 60% of SFTs were outside of the pleura and histopathology confirmed that SFTs were well-established and generally benign. These SFTs were strongly immunopositive for CD34, and CD99 was most positively expressed; Ki67 was expressed in 10% of SFTs. Tumors were immunonegative for Bcl-2, SMA, CKpan, S-100, and EMA protein (Table 1).

CT or MRI helps to identify SFTs and often reveals proliferation of fibrous tissues as well as tumor and adjacent tissue details to facilitate decisions for surgical tumor removal.^17^ Still, CT and MRI features of SFTs are nonspecific, revealing well-defined and often lobulated soft tissue space-occupying masses that displace adjacent anatomic structures.^18,19^ For our 20 cases, 12/20 patients with SFTs who were diagnosed in our single hospital were located in extrapleural sites (60%). SFTs of different sizes had different features according to CT and MRI.

In the field of medical diagnosis, receiver operating characteristic (ROC) curves have become the standard tool for assessment of predictive accuracy. In this study, we used ROC curve to determine the best cut-off values of the standard for sensitivity and specificity imaging features of SFTs. With the ROC curve analysis, the area under the curve (AUC) was 0.95 (95% confidence interval, 2.98–3.03). The larger SFTs (≥3.0 ± 0.203 cm) were heterogeneous masses with patchy, necrotic foci, and smaller SFTs (≤3.0 ± 0.203 cm) were homogeneous masses.

Unenhanced CT images revealed larger SFTs were isodense relative to muscle and some contained hypodense areas representing necrotic, myxoid, or cystic changes. With MRI, T2WI of hypointense lesions were related to hypercellularity and abundant collagenous stroma. Hyperintense lesions were relative to necrotic, myxoid, or cystic changes. Enhanced CT or MRI revealed mild to marked heterogeneous contrast uptake correlating with hypocellularity or hypervascular areas, which is consistent with previous reports.^20,21^

Mixed patterns were common in larger SFTs and smaller SFTs were isodense or isointense in both on unenhanced and enhanced CT and MRI.^22^ Unlike other reports,^23,24^ we observed no calcification in any of the 20 cases in our study, suggesting that this is uncommon in SFTs.

Most SFTs are benign but a few have histologically malignant features such as increased cellularity with crowded/overlapping nuclei, cellular pleomorphisms, and a mitotic count of >4 dividing cells per 10 high-power fields.^13^ CT and MRI confirm that malignant mass edges had infiltrating growths and hemorrhages or necrosis and lung metastasis were also observed.^25^ Finally, clinical outcomes for malignant SFTs are not always poor.^26^

To our knowledge, reports of SFTs from numerous extrapleural locations are most common in the literature and these include localization to the retroperitoneal, intrameningeal, or pelvic space; the thyroid or mammary gland; the cervical spine, the orbital space, and in the extremities.^27,28^ Of identified 20 patients with SFTs, 60% were extrapleural.

The findings of SFTs on CT and MRI are generally nonspecific for pathognomonic diagnosis. Differential diagnosis of STF includes localization to the pleura for mesotheliomas, to the brain for dural-based meningiomas or hemangiopericytomas, to the abdomen for interstitialomas, and to the kidney for angiomyolipomas, and so on.^29–31^ Diagnoses should be considered along with evidence from CT and MRI, histopathological findings, and CD34 immunoreactivity data. Surgical resection is the best long-term cure and helps to establish a confirmatory pathologic diagnosis for SFTs.

Like in every study, our study also has some limitations. First, in such a long-time survey and using different imaging techniques, it is difficult to maintain consistency. So, we have not measured the CT or MRI intensities and have not done any quantitative comparison because we have found that the SFTs have not any typical feature of MRI intensities and the different scanners with 1.5 and 3T. However, the typical dates such as the size of tumor at CT or MRI measured at the contrast-enhanced images of each transaxial section were averaged for each patient and descriptively compared among patients. Second, this is a retrospective study with SFTs patients who underwent MRI in our single hospital with a period of time and further research in a larger population is required to refine the imaging findings.

## CONCLUSION

We describe variable imaging findings for SFTs from our medical center. SFTs are rare tumors that occur postperitoneally and in organs such as the kidney and pancreas. Approximately 60% of SFTs are not within the pleura. With the ROC curve analysis, CT and MRI findings of mixed patterns of SFTs > 3.0 ± 0.203 cm at the greatest axis and SFTs < 3.0 ± 0.203 cm at the greatest axis are isodense with contrast enhancement. Thus, radiologists should be aware of potential diagnoses of SFTs. A final diagnosis of SFT can be established by histological examination of tissue taken from percutaneous biopsy or surgery, and surgery is the first treatment choice.
